# Supplementary material for: In situ and transcriptomic identification of microglia in synapse-rich regions of the developing zebrafish brain
Source: Nat Commun. 2021 Oct 8;12:5916. doi: 10.1038/s41467-021-26206-x (PMC8501082; doi:10.1038/s41467-021-26206-x)
Supplement: Supplementary file 1 — Supplementary Information [file 41467_2021_26206_MOESM1_ESM.pdf]

Supplementary figures, reagents, and software packages:

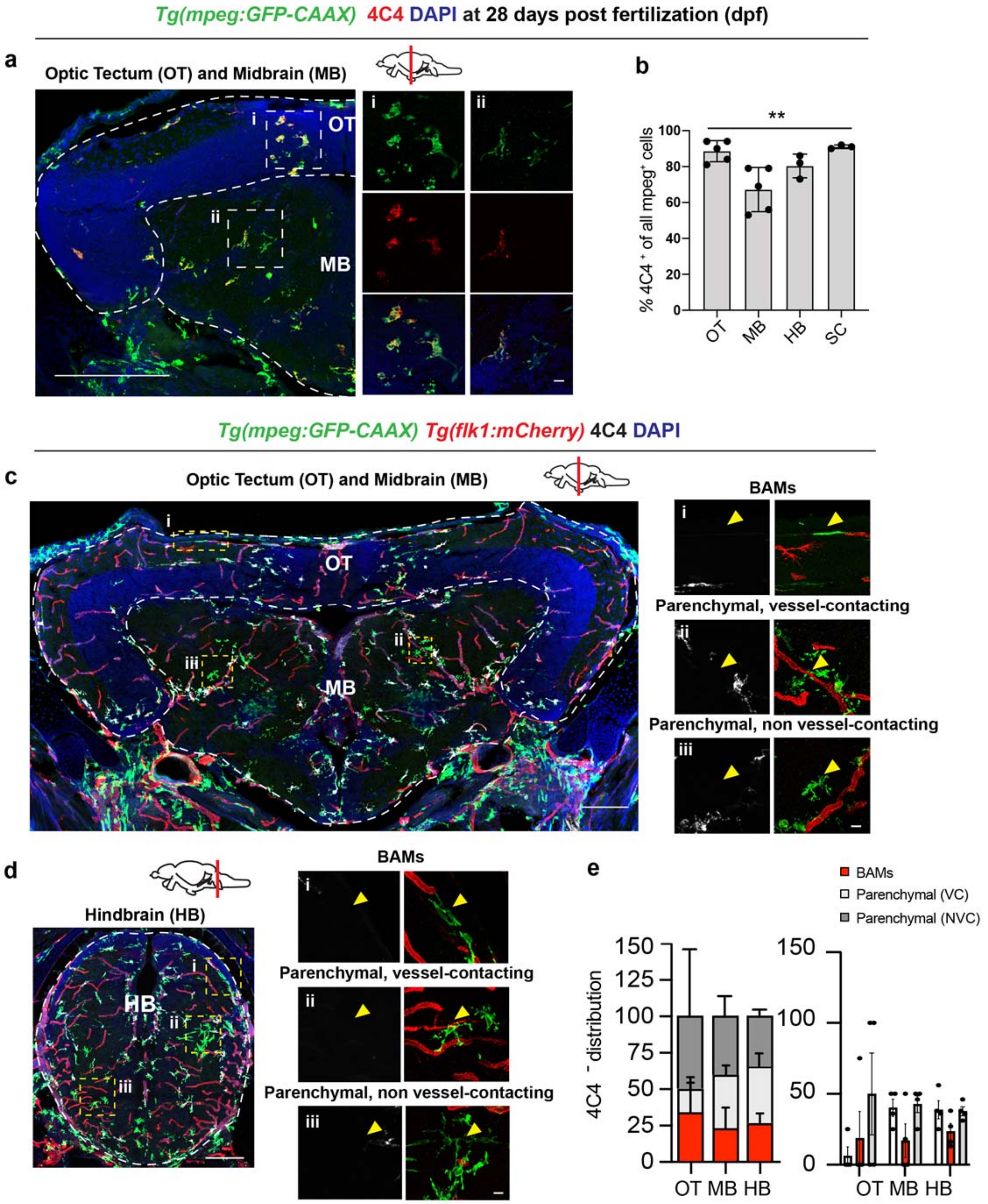

Figure S1. Characterization of 4C4 positive and negative myeloid cells, supplemental to Figure 1.

(a) Representative images of OT and MB brain regions using the pan-myeloid reporter line *mpeg1.1:*GFP-CAAX and the commonly used antibody 4C4 to label presumptive microglia at 28

days post fertilization (dpf). Scale: 100 µm. Optic tectum (OT), midbrain (MB), and hindbrain (HB). Insets of boxed areas show separate channels (top) and merged image (bottom) for each respective region. Scale: 10 µm. All images are representative of the n=3 replicates.

(b) Quantification of percent of *mpeg1.1:*GFP-CAAX$^+$ population also 4C4 positive. Dots represent individual fish, data are mean ± SD. One way ANOVA; ** p = 0.0047.

(c-d) Characterization of 4C4-negative myeloid cells. Representative images using the pan-myeloid reporter line Tg(*mpeg1.1:GFP-CAAX*) crossed to vascular reporter line Tg(*flk1:mCherry*), stained with the 4C4 antibody at 30 dpf. i) Insets highlighting *mpeg1.1:* GFP-CAAX$^+$ 4C4 negative border associated macrophages (BAMs) at the brain surface, ii) insets of representative *mpeg1.1:* GFP-CAAX+ 4C4-negative ramified parenchymal cell contacting a blood vessel; note, these cells lacked elongated perivascular macrophage morphology seen in mammalian PVMs. iii) Insets highlighting a *mpeg1.1:*GFP-CAAX$^+$ 4C4-negative, ramified, parenchymal, non-vessel contacting cell. Scale: 10 µm. All images are representative of the n=3 replicates.

(e) Distribution of the 4C4-negative *mpeg1.1:*GFP-CAAX$^+$ cell population across the three morphologically and regionally defined subsets shown in C-D. Dots represent individual fish (n=4), data are mean ± SEM. Scale: 100 µm. Optic tectum (OT), midbrain (MB), and hindbrain (HB).

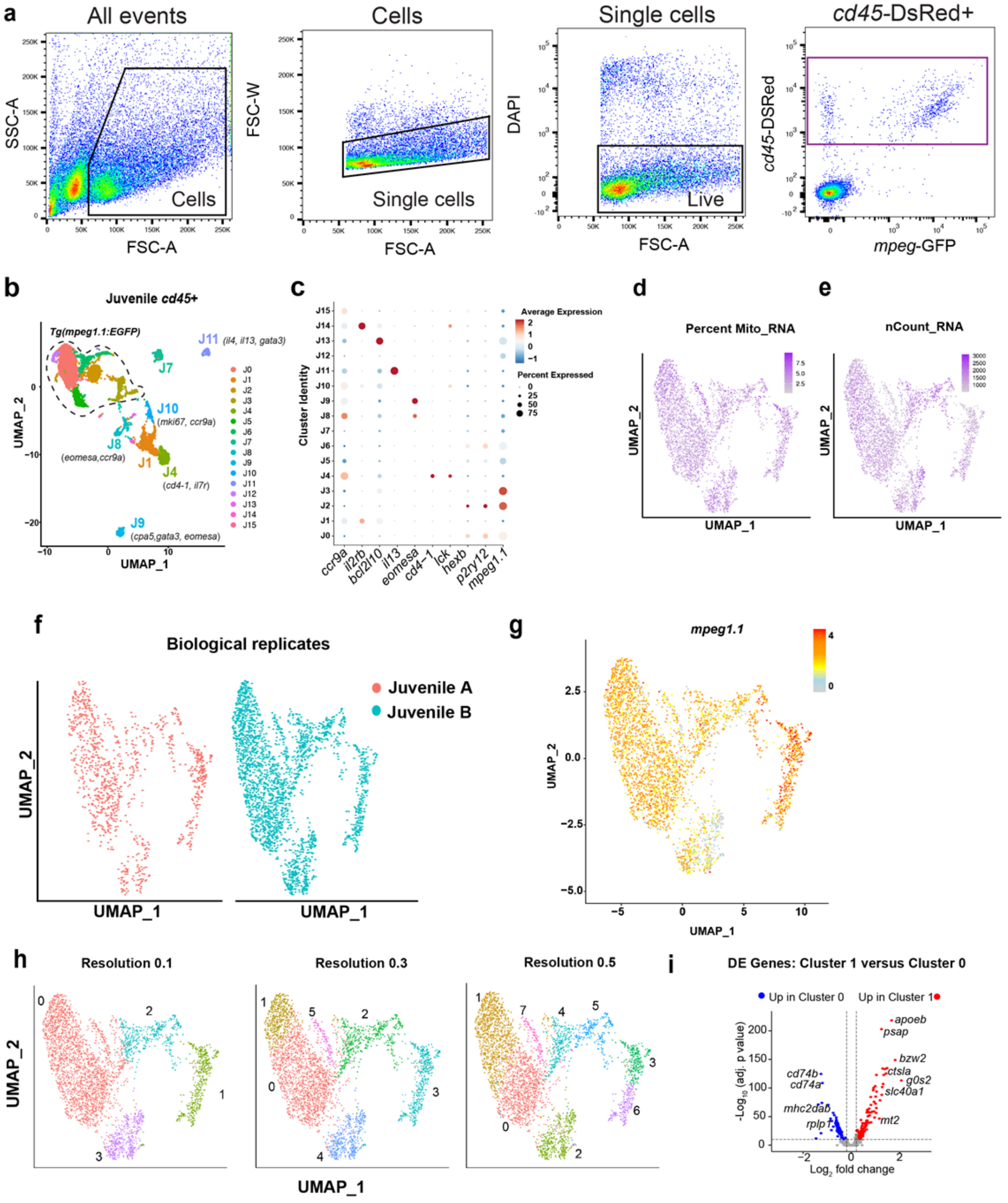

Figure S2. Gating hierarchy and quality control of single cell sequencing data, supplemental to Figure 2.

(a) Gating strategy to isolate *cd45* positive cells for single-cell RNA sequencing data in Figure 2 a-g. The violet gate represents the total *cd45*-DsRed^{+} population that was sequenced.
(b) Unsupervised clustering of juvenile *cd45* positive cells, replicated from Fig. 2b for reference.
(c) Select enriched genes in *cd45+mpeg1.1*$^{-}$ immune cell subsets calculated with the MAST DE algorithm in Seurat. Size represents percent of cells expressing each gene while color represents normalized and scaled gene expression compared to all clusters; decreased expression: blue, expression unchanged: white, increased expression: red.
(d-e) Feature plot showing distribution of mitochondrial RNA content and genes recovered (nCount) per cell.
(f) Comparison of independent biological replicates of *mpeg1.1*$^{+}$ cells pooled in Fig. 2c.
(g) UMAP plot highlighting levels of *mpeg1.1* expression the *mpeg1.1*$^{+}$ population from 2c.
(h) UMAP plots showing the effects of changing the clustering resolution on unsupervised clustering in Seurat with the FindClusters function. Clustering resolutions 0.1, 0.3, and 0.5 are shown.
(i). Volcano plot of differentially expressed genes between clusters JM1 (cluster 1) and JM0 (cluster 0) using a clustering resolution of 0.3. The MAST DE algorithm in Seurat was used to calculate log fold changes. Thresholds represented by dotted lines were set to adjusted p value $<10^{-10}$, log(2) fold change > 0.2.

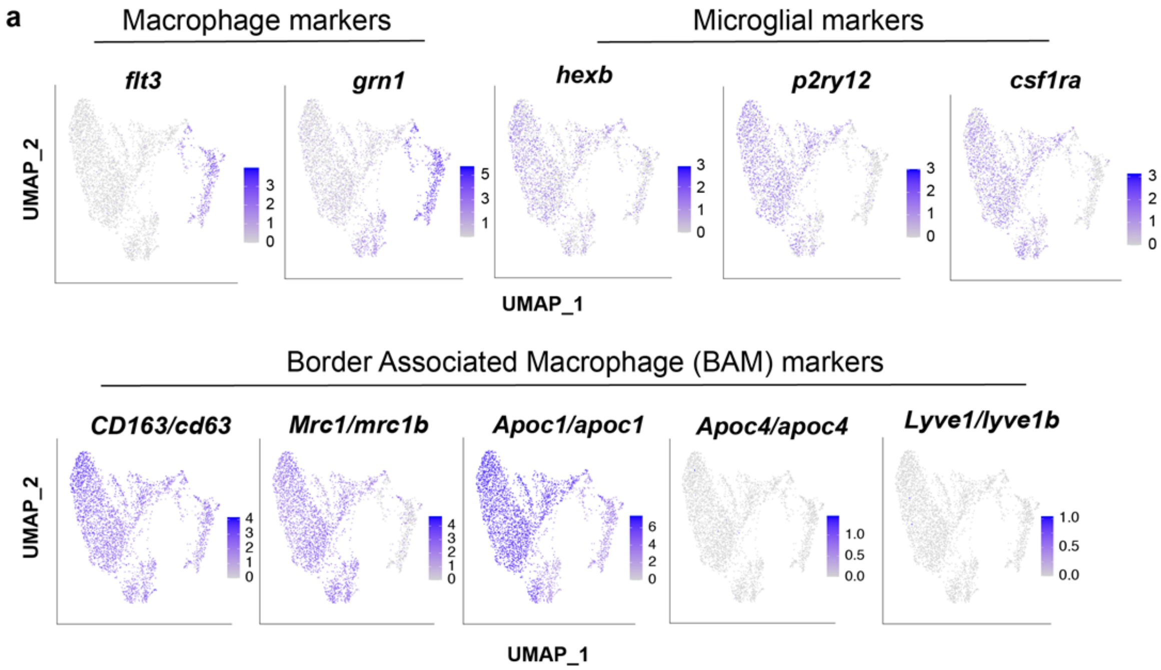

Figure S3: Feature plots of macrophage, microglia, and mammalian BAMs, supplemental to Figure 2.

(a) Feature plots from juvenile single-cell RNA dataset from Fig. 2C, showing canonical macrophage and microglia markers (top), and several proposed mammalian border associated macrophage (BAM) signature genes[39,56] with known homologs in fish (labels indicate mammalian/zebrafish homologs, bottom).

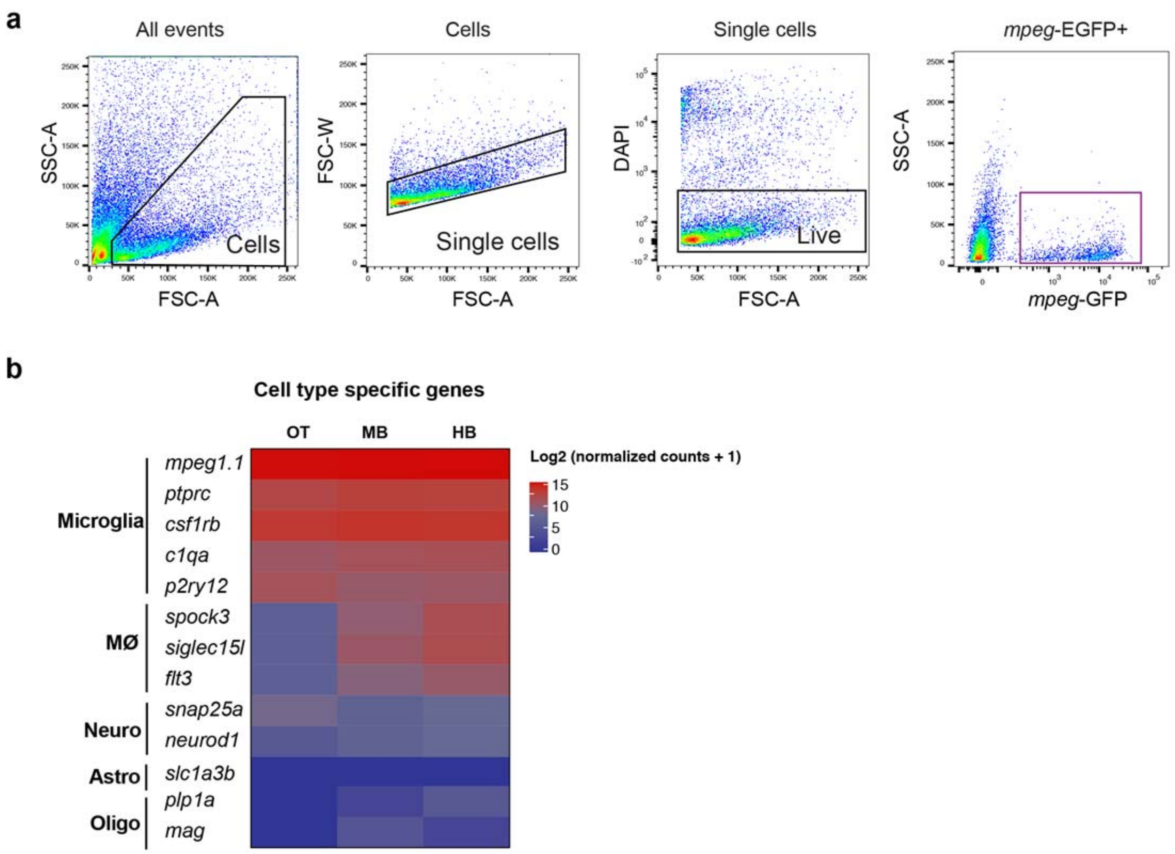

Figure S4. Gating strategy and quality control of bulk sequencing data, supplemental to Figure 3.

(a) Gating strategy to isolate *mpeg1.1*-EGFP$^+$ myeloid cells for region-specific bulk sequencing data in Figure 3A-C. The violet gate represents the *mpeg1.1*-EGFP$^+$ population that was sequenced.

(b) Heatmap of selected microglia, macrophage, neuronal, astrocyte, and oligodendrocyte genes.

Optic tectum (OT), midbrain (MB), and hindbrain (HB). Log-normalized counts generated with DESeq2.

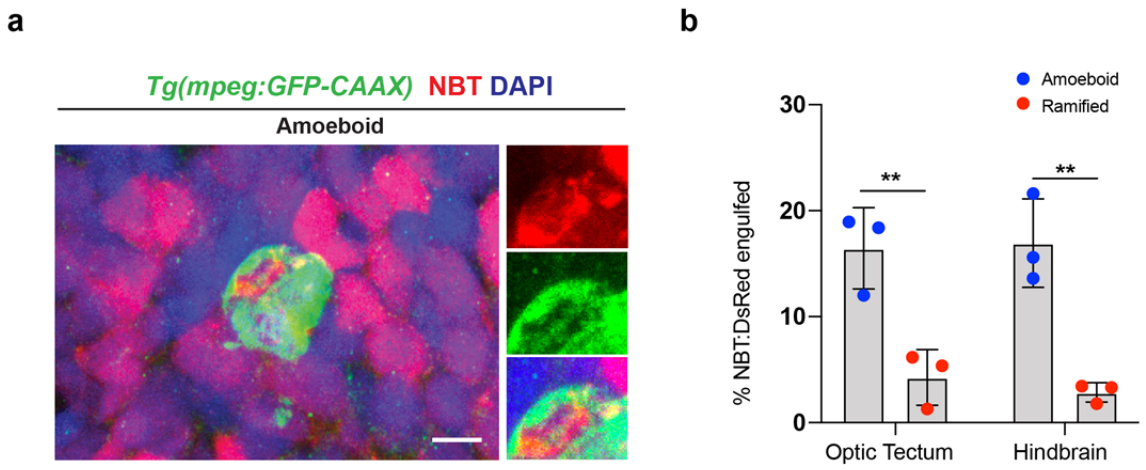

Figure S5. Neuronal engulfment in the OT and HB at 28 dpf, supplemental to Figure 4.

(a-b) Representative image and quantification of *NBT*:DsRed neuronal bodies within ramified or amoeboid microglia (Sphericity > .6) from OT and HB brain regions. Two separate t-tests, $^{**}p < 0.01$, n=3 microglia per fish; n=3 represented as mean values. Scale bar: 10 µm. All images are representative of the n=3 replicates.

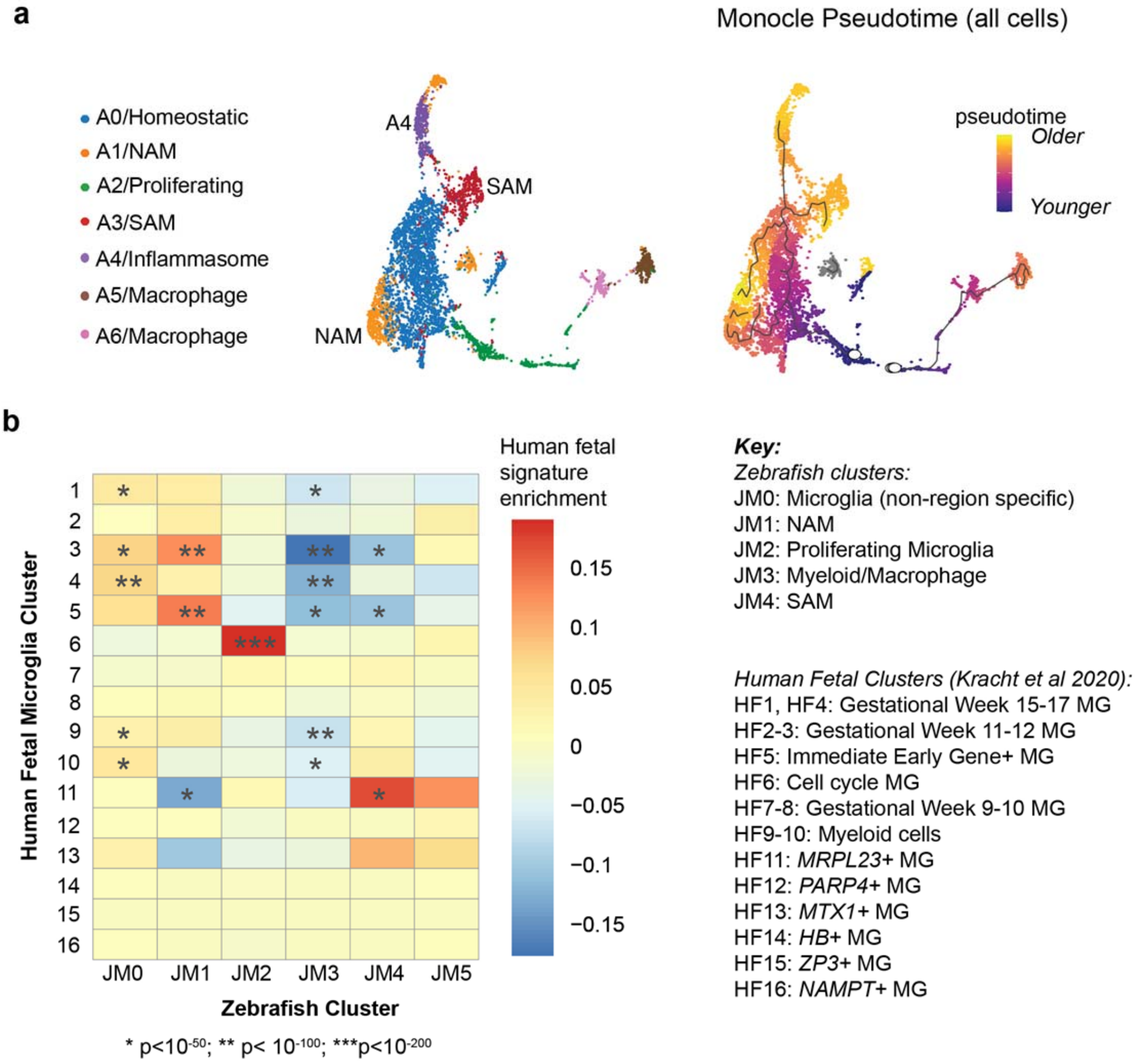

Figure S6. Human fetal microglia (Kracht et al., 2020) comparison to juvenile zebrafish microglia and pseudotime analysis, supplemental to Figure 4.

(a) Pseudotime analysis with Monocle 3[48] on both juvenile and adult *mpeg1.1* positive cell populations. Left UMAP plot is colored by cluster, right colored by estimated pseudotime using dividing cells as a starting point.

(b) Heatmap comparing juvenile zebrafish microglial clusters to a single-cell human fetal microglia dataset (Kracht et al 2020, table S3)[49]. Cells are colored by signature enrichment (estimated increase in average eigengene expression calculated with the AddModuleScore function in Seurat) in the listed zebrafish cluster compared to all other zebrafish clusters. (Two-sided Wilcoxon Rank-Sum Test, *$p<10^{-50}$, **$p<10^{-100}$, ***$p<10^{-200}$).

List of key reagents and software packages

REAGENT or Software package	SOURCE	IDENTIFIER
Antibodies		
Chicken anti-GFP 1:1000	Aves Labs	Cat#GFP-1020, RRID:AB10000240
Rabbit anti-DsRed 1:1000	Takara Bio	Cat#632496;RRID:AB-_10013483
Mouse anti-synaptic vesicle glycoprotein 2A (SV2) 1:500	DHSB	RRID: AB_2315387
Rat anti-BrdU 1:500	Abcam	Cat#ab6326;RRID:AB_305426
Mouse anti-4C4 1:200	Gift from Hitchcock Lab	Cat#92092321;RRID:AB_10013752
Anti-Digoxigenin-AP, Fab Fragments 1:500	Roche	Cat#11093274910,RRID:AB_2734716
Alexa Fluor 488 goat anti-chicken 1:500	Thermo Fisher	Cat#A-11039;RRID:AB_2534096
Alexa Fluor 555 goat anti-mouse 1:500	Thermo Fisher	Cat#A-21422, RRID:AB_141822
Alexa Fluor 555 goat anti-rabbit 1:500	Thermo Fisher	Cat# A-21429;RRID:AB_2535850
Alexa Fluor 647 goat anti-mouse 1:500	Thermo Fisher	Cat# A-21235; RRID:AB_2535804
Alexa Fluor 647 goat anti-Rat 1:500	Thermo Fisher	Cat# A-21248; RRID:AB_2535816
Critical Commercial Assays		
Chromium single cell gene expression platform, version 3	10x Genomics	Library and gel bead kit-V3, 120267:Chip B kit:1000009
SIGMAFAST Fast Red TR/Naphthol AS-MX Tablets	Sigma-Aldrich	C# F4648
ProSense 680 Fluorescent Imagining Agent	PerkinElmer	C#NEV10003
Deposited Data		
Single cell RNA-Sequencing of microglia from juvenile and adults	Gene Expression Omnibus	GSE164771
Bulk RNA-sequencing of microglia from OT, MB, HB brain regions	Gene Expression Omnibus	GSE164772
Experimental Models: Organisms/Strains		
Zebrafish*: Tg(mpeg1.1:EGFP)*	[57]	ZFIN: ZDB-ALT-0000
Zebrafish*: Tg(cd45:DsRed)*	[33]	
Oligonucleotides		
cd74a ISH primers,	In house	F: TAATACGACTCACTATAGGGGGACAGGAGAAACTCAAGG;R: AATTAACCCTCACTAAAGGGC

		CATCCCAAACAACATGC
ctsba ISH primers,	In house	F: TAATACGACTCACTATAGGGATGCAAGAGAGCAGTGG; R: AATTAACCCTCACTAAAGGGAAGTCCAATGAGCAGGTC
Seurat (Version 3.1.4)	[58]	https://satijalab.org/seurat
STAR (Version 2.5.4b)	[59]	https://github.com/alexdobin/STAR/releases
DESeq2 package (Version 4)	[60]	hhtp://www.bioconductor.org/packages/release/bioc/html/DESe2.html
Harmony (Version 1.0)		
ImageJ	NIH	RRID: SCR_003070
Imaris	Oxford Instruments	RRID: SCR_007370
Prism	GraphPad	RRID: SCR_002798

Supplemental dataset excel files:

Data S1: Pan hematopoietic single cell clusters in 28 dpf zebrafish brain
Legend: Differentially expressed genes per cluster for all juvenile (28 dpf) zebrafish CD45+ cells, as shown in Fig. 2b, S2b. First tab includes all clusters; further tabs are subsets of the first tab for each individual cluster. Column 1 (“gene”) = gene name; column 2 (“p_val”) = unadjusted p-value calculated with the MAST test in Seurat. Column 3 (“avg_logFC) = average natural log fold change for that gene between the labelled cluster (column 7, “cluster”) and all other cells. Column 4 (“pct.1”) = fraction of cells in the labelled cluster expressing that gene. Column 5 (“pct.2”) = fraction of cells outside of the labelled cluster expressing that gene. Column 6 (“p_val_adj”) = Bonferroni adjusted p-value per gene. Column 7 (“cluster”) = cluster shown in UMAP plots referenced above. Filtered to show p_val_adj < 0.001, avg_logFC>0.2.

Data S2: Myeloid single cell clusters in 28 dpf zebrafish brain
Legend: Differentially expressed genes per cluster for all juvenile (28 dpf) myeloid (*mpeg1.1*+) cells, as shown in Fig. 2C. First tab includes all clusters; further tabs are subsets of the first tab for each individual cluster. Column 1 (“gene”) = gene name; column 2 (“p_val”) = unadjusted p-value calculated with the MAST test in Seurat. Column 3 (“avg_logFC) = average natural log fold change for that gene between the labelled cluster (column 7, “cluster”) and all other cells. Column 4 (“pct.1 [in cluster]”) = fraction of cells in the labelled cluster expressing that gene. Column 5 (“pct.2 [out of cluster]”) = fraction of cells outside of the labelled cluster expressing that gene. Column 6 (“p_val_adj”) = Bonferroni adjusted p-value per gene. Column 7 (“cluster”) = cluster shown in UMAP plots referenced above. Filtered to show p_val_adj < 0.001, avg_logFC>0.2.

Data S3: Adult and juvenile single cell clusters

Legend: Differentially expressed genes per cluster for all juvenile (28 dpf) and adult (12 months) myeloid (*mpeg1.1*+) cells, as shown in Fig. 2e. First tab includes all clusters; further

tabs are subsets of the first tab for each individual cluster. Column 1 (“gene”) = gene name; column 2 (“p_val”) = unadjusted p-value calculated with the MAST test in Seurat. Column 3 (“avg_logFC) = average natural log fold change for that gene between the labelled cluster (column 7, “cluster”) and all other cells. Column 4 (“pct.1 [in cluster]”) = fraction of cells in the labelled cluster expressing that gene. Column 5 (“pct.2 [out of cluster]”) = fraction of cells outside of the labelled cluster expressing that gene. Column 6 (“p_val_adj”) = Bonferroni adjusted p-value per gene. Column 7 (“cluster”) = cluster shown in UMAP plots referenced above. Filtered to show p_val_adj < 0.001, avg_logFC>0.2.

Data S4: Region specific myeloid cell profiling in 28 dpf zebrafish brain

Legend: Differentially expressed genes calculated from bulk sequencing data comparing three brain regions (tab 1: hindbrain (HB, lfc >0) vs midbrain (MB, lfc <0); tab 2: optic tectum (OT, lfc >0) vs hindbrain (HB, lfc <0); tab 3: optic tectum (OT, lfc >0) vs midbrain (MB, lfc < 0). Statistics were conducted using the DESeq2 package in R, and results were filtered to p_adj < 0.05. Column 1 (“Ensembl_ID”): gene-specific Ensembl ID; Column 2 (“Gene_ID”): gene name (if available); Column 3 (“baseMean”): mean normalized gene expression over both samples, corrected for size factors; Column 4 (“log2FoldChange”): Log base 2 fold change gene expression in the first listed brain region compared to the second listed brain region. Positive values indicate enrichment in the first listed brain region; Column 5 (“lfcSE”): log fold change standard error estimate; Column 6 (“stat”): Wald test statistic value for the gene; Column 7 (“pvalue”); p-value associated with the test statistic; Column 8 (“padj”): Benjamini-Hochberg (FDR < 0.05) corrected p-value.

Data S5: Differentially expressed genes between HB-enriched cluster JM4 and OT-enriched cluster JM1.

Legend: Differentially expressed genes between clusters JM4 and JM1, as shown in Fig. 4d. Column 1 (“gene”) = gene name; column 2 (“p_val”) = unadjusted p-value calculated with the MAST test in Seurat. Column 3 (“avg_logFC) = average natural log fold change for that gene between cluster JM4 and JM1. Positive values represent increases in cluster JM4 with respect to JM1, while negative values represent genes increased in cluster JM1 with respect to JM4. Column 4 (“pct.JM4”) = fraction of cells in cluster JM4 expressing that gene. Column 5 (“pct.JM1”) = fraction of cells in cluster JM1 expressing that gene. Column 6 (“p_val_adj”) = Bonferroni adjusted p-value per gene. Column 7 (“cluster”) = cluster shown in UMAP plots referenced above. Filtered to show genes with p_val_adj < 0.001, avg_logFC>0.2.
