# Supplementary material for: In situ and transcriptomic identification of microglia in synapse-rich regions of the developing zebrafish brain
Source: Nat Commun. 2021 Oct 8;12:5916. doi: 10.1038/s41467-021-26206-x (PMC8501082; doi:10.1038/s41467-021-26206-x)
Supplement: Supplementary file 3 — Description of Additional Supplementary Files [file 41467_2021_26206_MOESM3_ESM.docx]

**Description of Additional Supplementary Files**

**Title: Supplementary Data 1. Pan hematopoietic single cell clusters in 28 dpf zebrafish brain**

**Description:** Differentially expressed genes per cluster for all juvenile (28 dpf) zebrafish CD45+ cells, as shown in Fig. 2b, S2b. First tab includes all clusters; further tabs are subsets of the first tab for each individual cluster. Column 1 (“gene”) = gene name; column 2 (“p_val”) = unadjusted p-value calculated with the MAST test in Seurat. Column 3 (“avg_logFC) = average natural log fold change for that gene between the labelled cluster (column 7, “cluster”) and all other cells. Column 4 (“pct.1”) = fraction of cells in the labelled cluster expressing that gene. Column 5 (“pct.2”) = fraction of cells outside of the labelled cluster expressing that gene. Column 6 (“p_val_adj”) = Bonferroni adjusted p-value per gene. Column 7 (“cluster”) = cluster shown in UMAP plots referenced above. Filtered to show p_val_adj < 0.001, avg_logFC>0.2.

**Title: Supplementary Data 2. Myeloid single cell clusters in 28 dpf zebrafish brain**

**Description: Differentially expressed genes per cluster for all juvenile (28 dpf) myeloid (*mpeg1.1*+) cells, as shown in Fig. 2C.** First tab includes all clusters; further tabs are subsets of the first tab for each individual cluster. Column 1 (“gene”) = gene name; column 2 (“p_val”) = unadjusted p-value calculated with the MAST test in Seurat. Column 3 (“avg_logFC) = average natural log fold change for that gene between the labelled cluster (column 7, “cluster”) and all other cells. Column 4 (“pct.1 [in cluster]”) = fraction of cells in the labelled cluster expressing that gene. Column 5 (“pct.2 [out of cluster]”) = fraction of cells outside of the labelled cluster expressing that gene. Column 6 (“p_val_adj”) = Bonferroni adjusted p-value per gene. Column 7 (“cluster”) = cluster shown in UMAP plots referenced above. Filtered to show p_val_adj < 0.001, avg_logFC>0.2.

**Title: Supplementary Data 3. Adult and juvenile single cell clusters**

**Description: Differentially expressed genes per cluster for all juvenile (28 dpf) and adult (12 months) myeloid (*mpeg1.1*+) cells, as shown in Fig. 2e.** First tab includes all clusters; further tabs are subsets of the first tab for each individual cluster. Column 1 (“gene”) = gene name; column 2 (“p_val”) = unadjusted p-value calculated with the MAST test in Seurat. Column 3 (“avg_logFC) = average natural log fold change for that gene between the labelled cluster (column 7, “cluster”) and all other cells. Column 4 (“pct.1 [in cluster]”) = fraction of cells in the labelled cluster expressing that gene. Column 5 (“pct.2 [out of cluster]”) = fraction of cells outside of the labelled cluster expressing that gene. Column 6 (“p_val_adj”) = Bonferroni adjusted p-value per gene. Column 7 (“cluster”) = cluster shown in UMAP plots referenced above. Filtered to show p_val_adj < 0.001, avg_logFC>0.2.

**Title: Supplementary Data 4. Region specific myeloid cell profiling in 28 dpf zebrafish brain**

**Description:** Differentially expressed genes calculated from bulk sequencing data comparing three brain regions (tab 1: hindbrain (HB, lfc >0 ) vs midbrain (MB, lfc <0); tab 2: optic tectum (OT, lfc >0) vs hindbrain (HB, lfc <0); tab 3: optic tectum (OT, lfc >0) vs midbrain (MB, lfc < 0). Statistics were conducted using the DESeq2 package in R, and results were filtered to p_adj < 0.05. Column 1 (“Ensembl_ID”): gene-specific Ensembl ID; Column 2 (“Gene_ID”): gene name (if available); Column 3 (“baseMean”): mean normalized gene expression over both samples, corrected for size factors; Column 4 (“log2FoldChange”): Log base 2 fold change gene expression in the first listed brain region compared to the second listed brain region. Positive values indicate enrichment in the first listed brain region; Column 5 (“lfcSE”): log fold change standard error estimate; Column 6 (“stat”): Wald test statistic value for the gene; Column 7 (“pvalue”); p-value associated with the test statistic; Column 8 (“padj”): Benjamini-Hochberg (FDR < 0.05) corrected p-value.

**Title: Supplementary Data 5. Differentially expressed genes between HB-enriched cluster JM4 and OT-enriched cluster JM1.**

**Description: Differentially expressed genes between clusters JM4 and JM1, as shown in Fig. 4d.** Column 1 (“gene”) = gene name; column 2 (“p_val”) = unadjusted p-value calculated with the MAST test in Seurat. Column 3 (“avg_logFC) = average natural log fold change for that gene between cluster JM4 and JM1. Positive values represent increases in cluster JM4 with respect to JM1, while negative values represent genes increased in cluster JM1 with respect to JM4. Column 4 (“pct.JM4”) = fraction of cells in cluster JM4 expressing that gene. Column 5 (“pct.JM1”) = fraction of cells in cluster JM1 expressing that gene. Column 6 (“p_val_adj”) = Bonferroni adjusted p-value per gene. Column 7 (“cluster”) = cluster shown in UMAP plots referenced above. Filtered to show genes with p_val_adj < 0.001, avg_logFC>0.2.
